# Leveraging Network Science for Social Distancing to Curb Pandemic Spread

**DOI:** 10.1109/ACCESS.2021.3058206

**Published:** 2021-02-09

**Authors:** Satyaki Roy, Andrii Cherevko, Sayak Chakraborty, Nirnay Ghosh, Preetam Ghosh

**Affiliations:** 1 Department of GeneticsUniversity of North Carolina Chapel Hill NC 27515 USA; 2 Department of Computer ScienceVirginia Commonwealth University 6889 Richmond VA 23284-3019 USA; 3 Department of CSTIndian Institute of Engineering Science and Technology 30130 Shibpur 711103 India

**Keywords:** Social distancing, network science, clustering, optimization, homophily

## Abstract

COVID-19 has irreversibly upended the course of human life and compelled countries to invoke national emergencies and strict public guidelines. As the scientific community is in the early stages of rigorous clinical testing to come up with effective vaccination measures, the world is still heavily reliant on social distancing to curb the rapid spread and mortality rates. In this work, we present three optimization strategies to guide human mobility and restrict contact of susceptible and infective individuals. The proposed strategies rely on well-studied concepts of network science, such as clustering and homophily, as well as two different scenarios of the SEIRD epidemic model. We also propose a new metric, called contagion potential, to gauge the infectivity of individuals in a social setting. Our extensive simulation experiments show that the recommended mobility approaches slow down spread considerably when compared against several standard human mobility models. Finally, as a case study of the mobility strategies, we introduce a mobile application, *MyCovid*, that provides periodic location recommendations to the registered app users.

## I. Introduction

COVID-19 has had an indelible imprint on human life and upended public health standards and social and economic order [1]. Nearly 1.7 million people have been reported dead worldwide by December 2020. However, the actual death numbers are likely to be greater due to want of testing, reporting and problems identifying cause of death [2], as some countries consider hospital deaths, while others factor in deaths at homes. The unprecedented mass hysteria surrounding this ongoing pandemic has bled into the businesses around the world, as millions of enterprises are projected to face extinction and families file for unemployment [3].

The road to a bonafide vaccination for COVID is long and arduous, particularly because they entail months, and sometimes years, of testing. However, the scientists are racing against time to come up with a vaccine by next year. At present 48 vaccines are under clinical trials on humans and nearly 88 being subjected to animal testing for best outcomes [4], making extensive social distancing our best bet to mitigate contagion. Health officials are continuing to harp on the role of physical distancing and self-quarantine measures towards flattening the curve [5]. There is a consensus in the scientific community regarding the possibility of contagion mitigation through the use of face coverings, personal hygiene, and by avoiding crowded and poorly ventilated places [6]. The clusters of infected populations were shown to be more likely in occupational or community settings, lending further credence to the significance of physical distancing [7]. The white collar jobs are relying on work-from-home and virtual communication, while the blue collar workers are being advised to abide by the 6 feet distance rule. There is a negative association between the number of days of lockdown and the reported COVID-19 cases per million [8].

### A. Related Works

The lack of prior knowledge on COVID-19 leaves the policymakers ill-equipped to design mitigation strategies. The research community of epidemiologists, clinicians and computer scientists are applying their expertise to seek out factors and their implications on contagion as well as economic downturn [9]. First, attempts are being made to apply machine learning (ML) to build prediction models on epidemiological and clinical data. Given existing clinical data, prediction models [10] and therapeutic approaches can help identify vulnerable groups [11], [12]. Epidemiologists are trying to identify spread dynamics of COVID-19. Holmdahl and Buckee [13] analyze the pros and cons of forecasting models that make predictions through curve fitting or mechanistic models, while supervised and unsupervised ML is helping trace the trends in infection dynamics [14]. Khan *et al.* used regression tree analysis, cluster analysis and principal component analysis on Worldometer infection count data to gauge the variability and effect of testing in prediction of confirmed cases [15]. Roy *et al.* perform regression analysis to identify pre-lockdown factors that affect the post-lockdown pandemic numbers [16] and topic modeling to find the least and worst affected economic sectors in the US [17].

Second, there have been efforts to modify the SEIRD model to study the effects of demography, immunity and social distancing on infection spread. SEIRD assumes that the susceptible person is exposed when he is in contact with an infected person (see Sec. II-A), implying that its accuracy depends on the correctness of knowledge of the epidemiological status of the individuals. However, with regard to COVID-19, (1) it may be hard to pinpoint when the susceptible person transitions to exposed; (2) a person is not deemed infected until tested positive. These factors can mislead SEIRD estimates. Gharakhanlou employed the SEIRD model to create an agent-based simulation to demonstrate the effects of social contact and propose mitigation measures to contain the spread of COVID-19 in Urmia city, Iran [18]. Bedi *et al.* proposed a modified SEIRD that considers a certain section of the exposed population to be infectious. They compare the COVID-19 projections made by their model on the different states of India against those from the Long Short-Term Memory (LSTM) model [19]. Ghanam *et al.* discuss a bayesian approach to estimate the parameters for the SEIRD model and quantify the impact of government intervention measures on infection spread [20]. Lattanzio *et al.* studied the relationship of lockdown and mobility in Lombardy and London as well as the ill-effects of flouting social distancing regulations [21]. Third, tracing contact using mobile apps has emerged as an approach to enforce physical distancing. Kretzschmar *et al.* evaluate the importance of timely contact tracing using a stochastic mathematical model with explicit time delays [22]. Ferretti *et al.* argue that app-based contact tracing together with virus-testing programs may help restrict further spread [23]. Ahmed *et al.* discuss the workings of the existing contact tracing apps based on proximity and duration of contact with infected individuals [24]. Campbell *et al.* designed a puzzle-game on top of an interactive learning environment, where players prepare for an outbreak on a social contact network and subsequently quarantine people to quell the epidemic [25]. Nadini *et al.* [26] created a mobile application that combines the features of *InfluenzaNet* [27] and *Flutracking* [28]. *InfluenzaNet* and *Flutracking* both perform an online survey to create a repository of symptoms of patients from geographic locations, with the objective to monitor spread and identify risk factors from symptoms.

### B. Contributions

Given a closed region (say, grocery store, queue at a bus stop, auditorium, stadium, etc.) where individuals are prone to high physical contact, we propose three optimization strategies to recommend new locations of individuals to curb pandemic spread. These optimizations operate on the same social contact network, yet vary on the basis of the underlying network science principle and epidemic model. While optimizations 1 and 2 minimize infection by eliminating contact and *network clustering* among susceptible and infected individuals in the SEIRD epidemic model, optimization 3 employs *homophily* on a modified SEIRD model (inspired from Bedi *et al.* discussed in Sec. I-A) where a population of the exposed asymptomatic (or untested) persons are spreaders of infection. Second, we introduce a new metric, called *contagion potential*, that quantifies the infectivity of an individual.

We carry out extensive simulation experiments on a small region in New York City to demonstrate that the three social distancing optimization strategies curb the spread of infection when compared against a random and two standard human mobility models, namely *least action trip planning* and *social network theory*. We also study how well these approaches preserve the network science principles of homophily and clustering as well as the effect of the epidemic parameters on the overall performance. Finally, we introduce a mobile application, called *MyCovid*, that presents a case study on the three optimization strategies and guide the registered users’ mobility to minimize contagion. With prior user permission, it can also create a repository of mobility traces and enable research on informing mobility during future outbreaks.

This paper is organized as follows. In Sec. 2 we cover preliminary concepts and system model. In Sec. 3, we present the three optimization strategies and the notion of contagion potential – a new metric to gauge infectivity. Sec. 4 has been dedicated to the experimental results, where we analyze the performance of the proposed optimization w.r.t. epidemic models, human mobility, scalability, parametric variations, etc., and introduce the features of the MyCovid app. Finally, we conclude the paper and discuss future works in Sec. 5.

## II. Preliminary Concepts and System Model

We first discuss the SEIRD epidemic model and key network science concepts used in the paper (viz., network clustering and homophily), followed by the experimental scenario.

### A. SEIRD Epidemic Model

We adapt the susceptible-exposed-infected-recovered-death (SEIRD) epidemic model [29]. The *susceptible* (S) class comprises individuals who are not exposed to the infection. Once exposed to infected individuals, they may transfer to the *exposed* (E) category, and this transition is controlled by a rate $\beta $. The E class are asymptomatic or untested individuals, who transition to the (tested) *infected* (I) class with probability $\sigma $. The individuals in $I$ transition to another state with a probability $\gamma $; this other state can be either *recovered* (R) or *dead* (D) with probabilities $1 - \alpha $ and $\alpha $, respectively, as shown below. Note that $\beta = \gamma \times R_{0}$, where $R_{0}$ is the basic reproduction number that has a median value of 3, but can be equal to 5.7 or even more as per previous literature [30], [31]. Thus, unlike, ($\gamma, \rho, \alpha $), $\beta $ is not a transition probability.

\begin{align*} S\xrightarrow [I]{\beta }&E \tag{1}\\ E\xrightarrow {\sigma }&I \tag{2}\\ I\xrightarrow {\gamma \times (1 - \alpha)}&R \tag{3}\\ I\xrightarrow {\gamma \times \alpha }&D\tag{4}\end{align*}

*Modified SEIRD Model:* We classify the exposed population $E$ into $E_{\hat {v}}$ and $E_{v}$ (i.e., $E = E_{\hat {v}} + E_{v}$), where the individuals in $E_{v}$ are the population of asymptomatic (or untested) individuals that do not transition to $R$ or $D$, while $E_{\hat {v}}$ are asymptomatic (or untested) individuals who transition to $R$ or $D$ states. Moreover, the susceptible individuals ($S$) may transition to exposed ($E$) category, if they come in contact with either infected ($I$) or spreaders or **v**ectors, i.e., $E_{v}$ individuals, as we show below:

\begin{align*} S\xrightarrow [I / E_{v}]{\beta }&E_{v} / E_{\hat {v}} \tag{5}\\ E_{\hat {v}}\xrightarrow {\sigma }&I \tag{6}\end{align*}

Eqs. 3, 4 are common for both SEIRD models. In case of modified SEIRD, we replace Eqs. 1, 2 of the original SEIRD with Eqs. 5 and 6. It is still difficult to identify the exposed individuals who act as vectors (i.e., $E_{v}$), and with more testing, some of them may be identified as infected.

### B. Key Network Science Concepts

The proposed social distancing optimization measures (delineated in Sec. III) are built upon two concepts of network science, particularly social network analysis, as follows.

#### 1) Clustering

It is a tendency of nodes to form tightly knit groups [32]. In an undirected graph $H(V, E)$, clustering coefficient of any node $u \in V$ is calculated as:

\begin{align*} \text {CC}(H,u) = \begin{cases} 0, & \text {if}~\delta (u) < 2 \\ \dfrac {2 \times t(u)}{\delta (u) \times (\delta (u) - 1)}, & \text {otherwise} \end{cases}\tag{7}\end{align*}

In the above equation $t(u)$ is the number of triangles node $u$ participates in and $\delta (u)$ is its degree. The *average clustering coefficient* ($\alpha $) of the undirected graph $H$ is given by –

\begin{equation*} \alpha (H) = \frac {1}{|V|} \sum _{u \in H} \text {CC}(H,u)\tag{8}\end{equation*}

**FIGURE 1.**
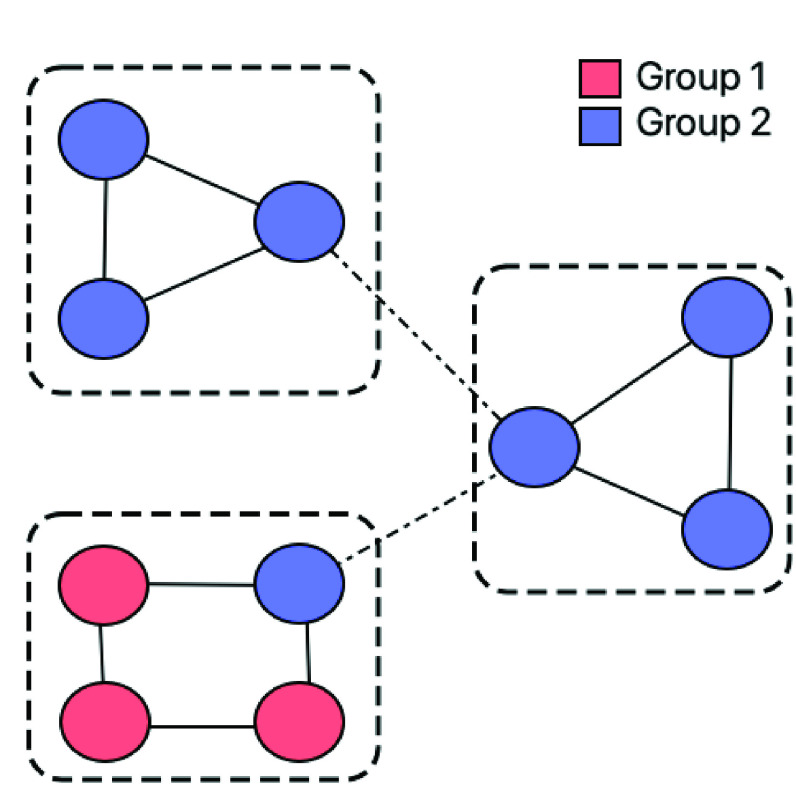
Clustering and Homophily. Three clusters demarcated by boxes, where a node belongs to either group 1 or 2 (differentiated using red and blue colors, respectively).

Given two *groups* (or labels) of nodes marked in red and blue, Fig. 1 shows three clusters demarcated in dotted boundaries. On a scale of 0 and 1, this network has $\alpha = 0.6$.

#### 2) Homophily

It is the tendency of a node to share links with other nodes with similar characteristics (i.e., groups or labels). Homophily (literally meaning *love of the same*) causes nodes to preferentially attach to similar nodes resulting in relationships like geographical proximity, friendship, etc. [33]. While network homophilicity is often verified using metrics such as dyadicity and heterophilicity [34], E-I index can be a simple measure for homophily [35]. It is calculated as the difference between between-group ties and within-group ties, divided by the total number of links in the network. Complete heterophily is quantified by an E-I index of 1, and complete homophily by a E-I score of −1. The network in Fig. 1 has E-I index = −0.6, suggesting that it is highly homophilic.

### C. Scenario

We simulate a region (of dimension $X \times Y$ square units) where mobile individuals $u \in V$ are confined. Each individual must initially belong to one of these epidemic states: susceptible or infected. From time to time, these individuals may come within some preassigned *contact threshold* $d$ (say, 6 ft. for COVID-19 [36]) of one another, allowing for the susceptible person to get exposed to the infection from an already infected person. The location of each person is known, and it is possible to capture the dynamics of physical contact between people by creating a contact graph (refer Sec. III), where the individuals are vertices (or nodes) and bidirectional links exist between any two persons within distance $d$ at any given time $t$. The system administrator may apply the proposed social distancing optimization approaches (discussed in Sec. III) on the time-varying contact graph at time $t$ and suggest a new position of a person within a distance threshold $\tau $ of its current location, in order to minimize infection spread. A person may or may not abide by the recommendation of the administrator.

### D. Human Mobility Model

#### 1) Least Action Trip Planning

This mobility model is based on the notion that humans tend to consider distance to be a crucial criterion for deciding the next destination, termed *waypoint* [37]. In other words, for any individual, the likelihood of choosing a certain waypoint is directly proportional to the proximity to his current location. Given a current waypoint $z$, the probability of selecting waypoint $w_{i}$ is:

\begin{equation*} p_{w_{i}} = \frac {\text {dist}(z, w_{i}))^{-a}}{\sum _{w_{j} \in \mathbf {W}} \text {dist}(z, w_{j}))^{-a}}\tag{9}\end{equation*}

Here, $\text {dist}(x, w^{i})$ is the Euclidean distance between $z$ and $w_{i}$, and $a$ is the weighing factor, a positive constant, that characterizes the preference to waypoints. If $a = 0$, a waypoint has an equal likelihood of being visited, while higher $a$ causes the closer waypoints to get a higher likelihood. For our experiment, we consider $a = 1.2$; this is based on the fact that LATP is shown to produce mobility traces that match the real GPS traces very well when $a$ lies between 1 and 3 [38].

#### 2) Social Network Based Mobility

People incorporate social interactions into their mobility decisions. A person visits areas where he may meet his kin. We implement the social network theoretic (SNT) model [39] for which we generate a (undirected) friendship network where the friends of a node are its 1-hop neighbor. Any node $u$ will choose its next waypoint with likelihood, on the basis of its the social affinity $A(w_{i})$, by the formula $p_{w_{i}} = \frac {A(u, w_{i})}{\sum _{j}^{|\mathbf {W}|} A(u, w_{j})}$. Given individual nodes $\eta (w_{i})$ located at waypoint $w_{i}$, the social affinity for waypoint $w_{i}$ having $\eta (w_{i})$ nodes, is calculated as follows:

\begin{align*} A (u, w_{i}) = \begin{cases} \dfrac {|v: v \in \eta (w_{i}) \& (u, v) \in V(Z)|}{|\eta (w_{i})|} & | \eta (w_{i})| > 0 \\ 0 & \text {Otherwise} \\ \end{cases}\end{align*}

In the above equation, $\frac {|v: v \in \eta (w_{i}) \& (u, v) \in V(Z)|}{|\eta (w_{i})|}$ is the ratio between the social affinity to the total population of $w_{i}$. *Social affinity* of a person to a zone is measured as the number of friends (i.e., 1-hop neighbors in the friendship graph) who are currently located in that given zone.

## III. Approach

Consider a set of individuals $u \in V$ are in moving in a closed region of dimension $X \times Y$ square units and intermittently coming in close contact. Given $T$ time slots, we create a contact graph $G_{t} = (V, \epsilon _{t})$ for $t \in T$, where the nodes $V$ are the individuals and edges $(u, v) \in \epsilon _{t}$ denote contact between (the individuals represented as) nodes $u, v \in V$ that are within a contact threshold of $d$ for at least a prespecified duration of time within the current time slot $t$. The neighbor-list of a node $u$, $n_{t}(u)$, is the set of individuals that are within distance $d$ at time $t$. Each individual $u$ must belong to exactly one of $S, E, I, R, D$ states, where $S \cup E \cup I \cup R \cup D = V$.

### A. Optimization Formulation

We discuss the intuition and formulation of the three social distancing optimization strategies. The first two approaches *opt-1* and *opt-2* apply the standard SEIRD model, while *opt-3* makes use of the modified SEIRD (refer Sec. II-A).

#### 1) Objective

Given the current location at time $t$, these optimizations calculate new locations for each individual to minimize contact that may potentially cause infection spread.

#### 2) Constraint

The optimizations have a common constraint (Eqs. 11. 13, 15) to ensure that the recommended location is not unrealistically far from current location of an individual. We define a *distance threshold* $\tau $ that dictates the maximum distance between the current and recommended locations.

### B. Approach 1

In the SEIRD model, a susceptible person may get exposed only upon contact with the infected individual, making the latter the only spreaders of infection in the contact network at time $t$, $G_{t}$. Hence, in *opt-1* we attempt to curb contagion by placing the nodes in a manner that we may minimize contact between the susceptible and infected people in the network.

\begin{align*}&\min \limits _{C_{t + 1}} \sum _{u \in S_{t} \cup E_{t}} \sum _{v \in I_{t}} f(u, v, G_{t}) \tag{10}\\&s.t. ~\text {abs}(C_{t + 1}(u) - C_{t} (u)) \leq \tau \tag{11}\end{align*}

Here $f(u, v, G_{t}) = 1$ if $(u, v) \in \epsilon _{t}$ and 0 otherwise, and $E_{t}$ denotes the set of exposed individuals at time $t$. We consider susceptible as well as exposed in objective (Expression 10), because one cannot differentiate between susceptible from the asymptomatic (or untested) exposed individuals, i.e., they are indistinguishable for the optimizer. Expression 11 ensures that recommended new location is within distance threshold $\tau $ from the present location ($C_{t}(u)$) of an individual $u$.

### C. Approach 2

**FIGURE 2.**
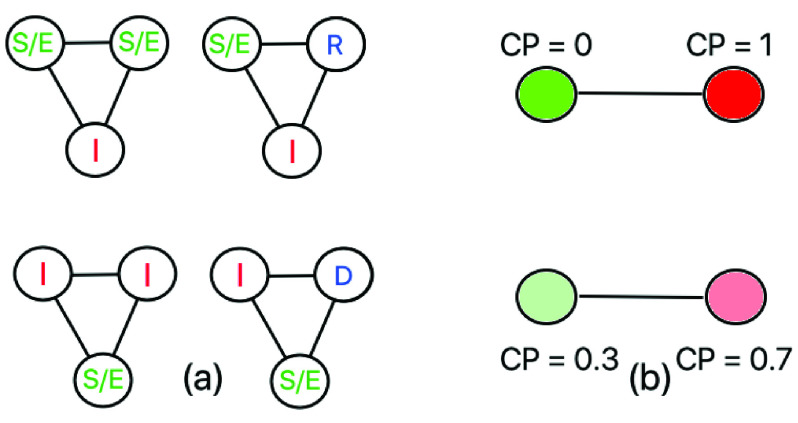
Social distancing optimizations. (a) the four triangular configurations involving at least one susceptible (or exposed) and one infected individual, (b) links between susceptible ($\text {CP} = 0$) and infected ($\text {CP} = 1$) individuals are eliminated in *opt-1* (top), whereas links showing a high difference in CP are removed in *opt-3* (bottom).

We expect the clusters in $G_{t}$ to have higher contact leading to greater contagion. Recall from Sec. II-B1, the clustering tendency of a node is proportional to the number of triangles it participates in. In this approach, we attempt to minimize the triangles involving at least 1 susceptible/exposed and 1 infected individual. Fig. 2a shows the four triangle configurations, that the following optimization eliminates.

\begin{align*}&\min \limits _{C_{t + 1}} \sum _{u \not \in R, D} \sum _{v \not \in R, D; v > u} \sum _{w \not \in R, D; w > v} \delta (u, v, w, G_{t}) \tag{12}\\&s.t. ~abs(C_{t + 1}(u) - C_{t} (u)) \leq \tau \tag{13}\end{align*}

Here $\delta (u, v, w, G_{t}) = 1$ if the following conditions hold:

1) $(u, v), (v, w), (u, w) \in \epsilon _{t}$, and
2) $u \in S_{t}/E_{t} || v \in S_{t}/E_{t} || w \in S_{t}/E_{t}$ and $u \in I_{t} || v \in I_{t} || w \in I_{t}$

$\delta (u, v, w, G_{t}) = 0$ otherwise.

Exp. 12 invokes function $\delta $ to minimize the occurrence of triangles with at least one susceptible/exposed and infected.

### D. Approach 3

In the modified SEIRD model (discussed in Sec. II-A), we define the exposed population as $E = E_{v} + E_{\hat {v}}$, where the individuals in $E_{v}$ are exposed, yet act as vectors of infection. Since we have no definitive knowledge of the individuals in $E_{v}$, it becomes imperative to devise a metric to gauge the likelihood that a person may act as spreader.

#### 1) Contagion Potential (CP)

It is the ability of an individual to act as the spreader of infection. The instantaneous CP of $u$ (s.t., $u \not \in R, D$) at time $t$ is proportional to the number of infected people in its current neighborhood ($n_{t}(u)$):

\begin{align*} P_{t}(u) = \begin{cases} 0, & t = 0\\ 1, & t \geq 1, u \in I\\ \dfrac {\sum _{v \in n_{t}(u)} P_{t - 1} (v)}{M_{t}} & \text {Otherwise} \end{cases}\end{align*}

Here, $M_{t}$ is the maximum number of neighbors of any node at time $t$. The overall CP till time $T$, $Z_{T}$, is calculated as the mean over instantaneous values, as follows:

\begin{align*} Z_{t}(u) = \begin{cases} 0, & u \in R, D\\ 1, & u \in I\\ \dfrac {1}{T} \sum _{t = 0}^{T} P_{t}(u) & \text {Otherwise} \end{cases}\end{align*}

#### 2) Intuition

**FIGURE 3.**
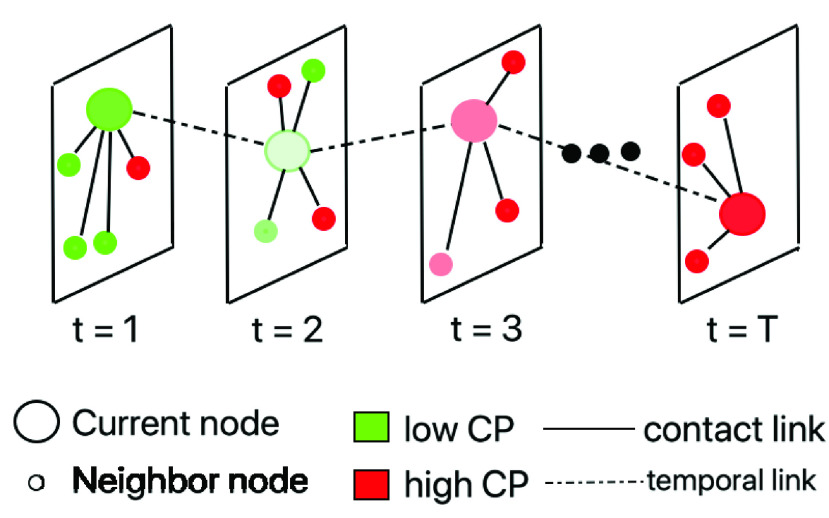
Evolution of contagion potential in *opt-3*. Each panel shows the location of individuals at a certain timepoint $t$ ($1 \leq t \leq 1$) and the colors green and red show their CP.

Considering the modified SEIRD model, in Fig. 3 we illustrate an individual node at different timepoints $t = 1, 2, \cdots, T$ with a different set of neighboring nodes. Initially the reference node (shown as a large circle) encounters very few infected neighbors (marked red) and is less likely to have contracted the infection and has a low CP (hence colored green). Over time, it transitions to high CP score upon contact with more high CP individuals. We would like to point out that CP can have significant implications on the accuracy of the traditional SEIRD model where we consider a binary infection status. Due to imperfections in the testing method or lack of testing, an infected individual is mistakenly deemed susceptible (or exposed). Under such a circumstance, CP, which gauges his encounters with other high CP or infected nodes, can serve as an alternative measure to quantify the likelihood of his being infected (and therefore, “infective”).

In the third optimization (*opt-3*), we calculate the CP for every individual not in the recovered or dead category. We intuit that contagion can be contained if the difference between the CP of any pair of individuals $u, v \not \in R, D$ in contact (i.e., $|\text {CP}(u) - \text {CP}(v)|$) is low. In other words, high $|\text {CP}(u) - \text {CP}(v)|$ may imply that the individual with the lower CP (say $u$) may contract the infection from $v$. Going back to the discussion in Sec. II-B2, if two individuals having same CP are considered to belong to the same groups (and vice versa), the current optimization attempts to create a homophilic network by eliminating links between individuals with disparate CP (see Expr. 14). In other words, we put nodes with similar CPs into the same groups and minimize edges between different groups having dissimilar CPs.

\begin{align*}&\min \limits _{C_{t + 1}} \sum _{(u, v) \in E_{t}, u, v \not \in R, D} |Z_{T} (u)\,\,- Z_{T}(v)| \tag{14}\\&s.t. ~abs(C_{t + 1}(u) - C_{t} (u)) \leq \tau \tag{15}\end{align*}

Above expression is a generalization of *opt-1* where a person holds a binary status of infected or not infected (as shown in Fig. 2b top). In effect, *opt-3* turns the infection status into a continuous variable in range $[{0, 1}]$ (see Fig. 2b bottom).

## IV. Result

We create a simulation environment in Python to validate the proposed methods. Let us discuss the experimental results in the following subsection: (1) effect of infection spread and contagion potential, (2) clustering and homophily, (3) parametric variations, (4) scalability, (5) human mobility, (6) *MyCovid* app, and (7) effects of flouting recommendations.

**TABLE 1:** Default Parameter Values

Parameter	Notation	Value
Number of iterations	–	5
Population size	$n$	100
Simulation area	$X, Y$	100 ft, 100 ft
Simulation duration	$T$	100 minutes
SEIRD parameters [30]	$\sigma, \gamma, \alpha, \beta $	$0.25, 0.1, 0.05, 0.55$
Contact threshold	$d$	6 ft
Distance threshold	$\tau $	6 ft
No. of waypoints	–	40
Edge prob. friend. net.	$p(\text {edge}) $	0.1
LATP parameter	$a$	1.2

*Default Parameters:* We perform the experiments, each of duration 100 minutes, on a population of 100 individuals and contact rate $\beta = 0.55$. We plot the mean curve from 5 iterations, showing the *cumulative count*, which we measure as the sum of infected, recovered and dead individuals at any given time. To ensure fairness of comparison, the individuals have the same initial starting location and epidemic status in each run of the experiment. The contact threshold is $d = 6$ ft. and individuals move within distance threshold $\tau = 6$ ft. on an average at every minute. Strategies *opt-1*, *opt-2* and *opt-3* are run on SEIRD and modified SEIRD, respectively. Recall from our discussion in Sec. III-D1, contagion potential (CP) is calculated over a period of time. Hence, for the experiments on *opt-3* utilizing CP (on the modified SEIRD model), we follow random mobility till $T = 5$ minutes. This allows each individual to achieve a steady CP, before *opt-3* is invoked.

### A. Effect on Infection Spread and Contagion Potential

#### 1) Contagion Potential

**FIGURE 4.**
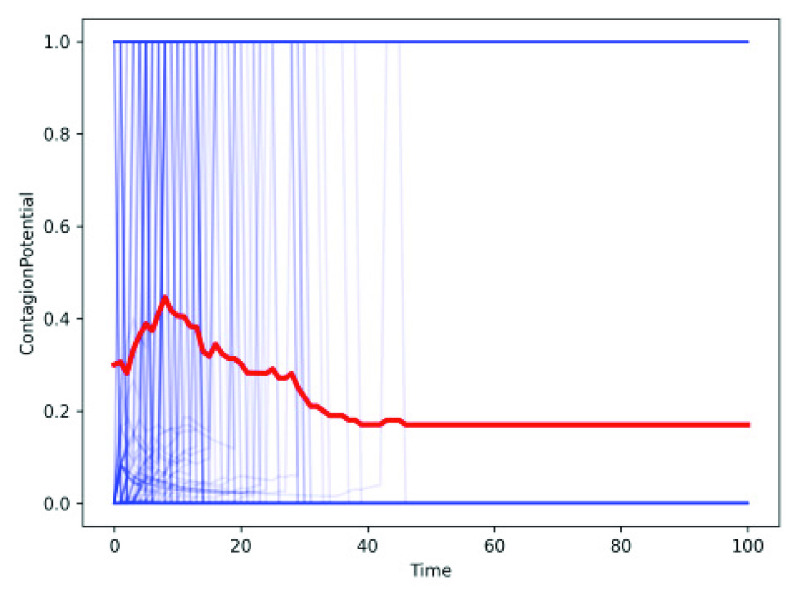
Contagion potential (CP) of an individual (shown in blue) and declining mean CP (shown in red) over time.

Recall from Sec. II-A, the modified SEIRD model allows a fraction of exposed individuals ($E_{v}$) to act as vectors. In Sec. III-D1, we introduce contagion potential (CP) (Sec. III-D1) to gauge how likely an exposed or infected individual is to act as the vector. Fig. 4 shows the CP of each individual in blue lines and the mean CP of the population in red in the modified SEIRD. Note, the mean CP declines over time as more people recover or die and their respective CPs become 0.

**FIGURE 5.**
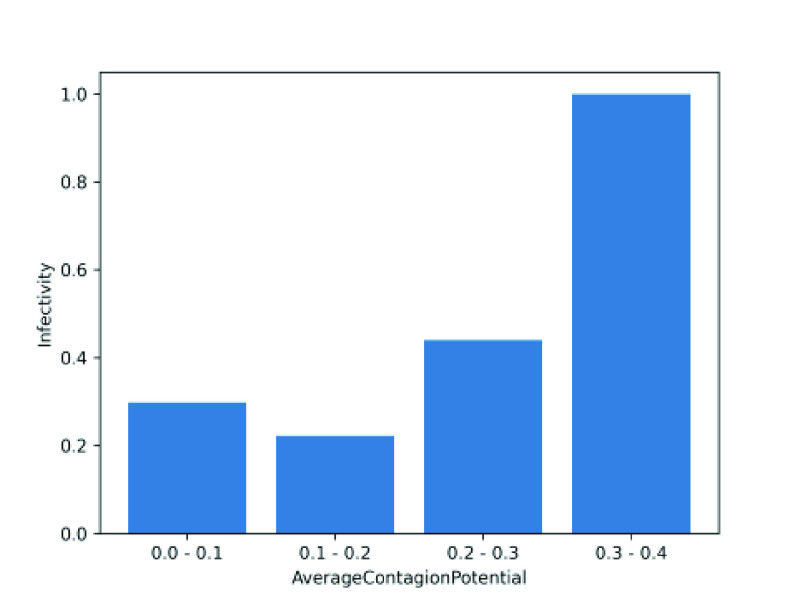
Change in infectivity with contagion potential.

We show the efficacy of CP by introducing another simple measure, called *infectivity*, measured as the ratio between the number of times an (exposed or infected) individual transmits infection to the total number of contacts with susceptible individuals. Fig. 5 depicts that the overall infectivity of an individual correlates with mean CP, suggesting that it can be an effective metric to identify latent spreaders of infection.

### B. Clustering and Homophily

**FIGURE 6.**
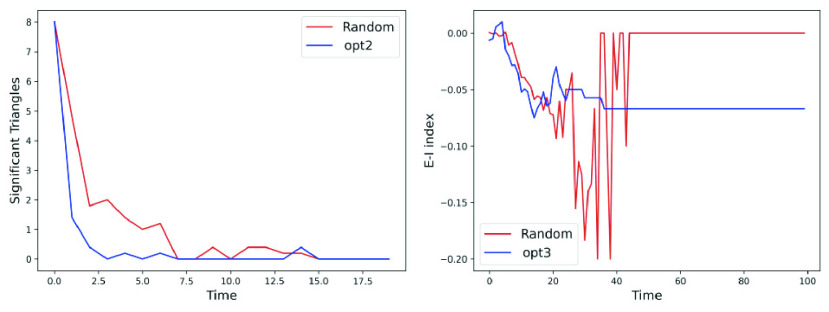
Verification of network science principles. Comparison of (a) number of clusters (or triangles) with at least one infected and one susceptible/exposed in the contact graph for random and *opt-2*, (b) E-I index for random and *opt-3*.

We discuss how the social distancing optimization *opt-2* minimizes significant clusters, while *opt-3* maximizes homophily in the contact network – both of which contribute towards containment of contagion (refer Sec. II-B2). Fig. 6a shows that *opt-2* has fewer number of significant clusters (i.e., triangles containing at least one infected and one susceptible/exposed). Similarly, to gauge whether *opt-3* achieves homophilic contact network with respect to CP, we assume two individuals $u, v \in V(G_{t})$ to be “similar” only if $|\text {CP}(u) - \text {CP}(v)| \leq 0.1$. In other words, if $\text {CP}(1) = 0.15$, it is deemed to similar to $v$ and $w$ with $\text {CP}(v) = 0.05$ and $\text {CP}(w) = 0.25$. We compare the E-I index (see Sec. II-B2) of random vs *opt3* at each time point. Fig. 6b shows that, not considering the recovered and dead, *opt-3* has lower E-I index (i.e., it creates homophilic networks with a lower difference in CP between the individuals) than random.

### C. Parametric Variations

We discuss in Sec. II-A that the modified SEIRD model allows a set of exposed individuals to also act as potential spreaders. In this section, we analyze how *opt-3* performs upon variation of the parameters in the SEIRD epidemic model, namely, (1) number of initial infected persons, (2) susceptible to vector transition probability $E_{v} (\beta)$ and (3) contact rate ($\beta $).

**FIGURE 7.**
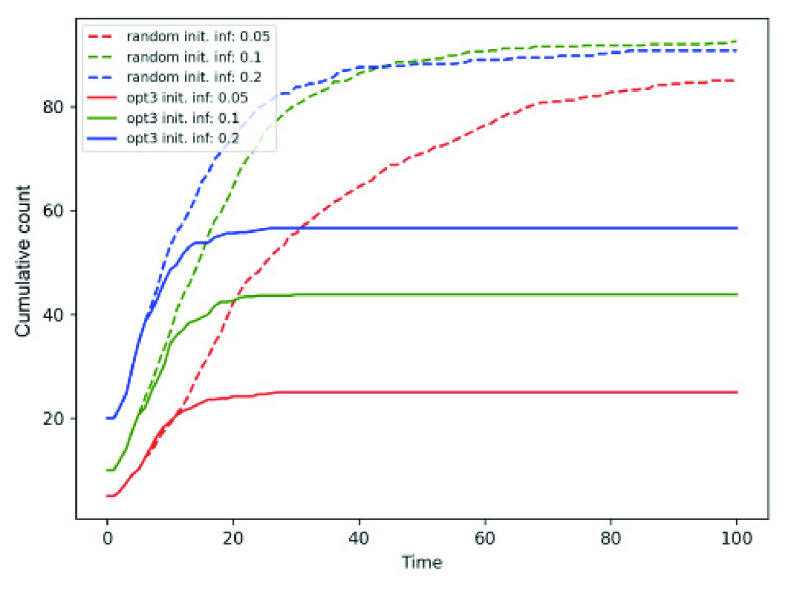
Cumulative count for varying number of initial infected individuals in random and *opt-3* approaches.

**FIGURE 8.**
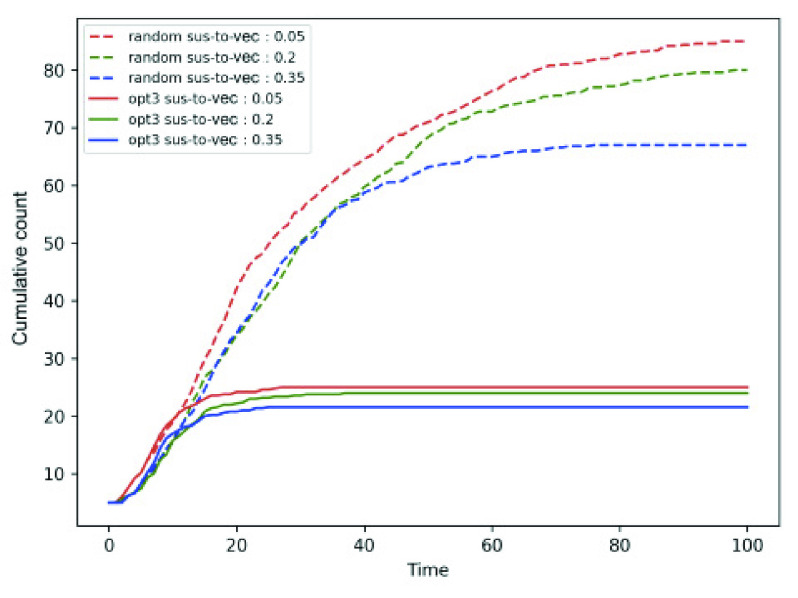
Cumulative count for varying susceptible to vector transition probability in random and *opt-3* approaches.

*Number of initial infected persons and susceptible to exposed vector* ($E_{v}$) transition probability. Fig. 7 shows that the cumulative count increases with initial infected population. Note that *opt-3* outperforms random mobility for all the three values of initial infected fraction $= 0.05, 0.1. 0.2$. Fig. 8 shows the variation in $\beta = 0.05, 0.2, 0.35$ for fixed initial infected fraction, where *opt-3* again outperforms random mobility. It is noteworthy that $\beta = 0.2$ curve (shown in green) shows lower cumulative $\beta = 0.05$. This is because, $\beta = 0.2$ causes a higher number of individuals to become part of the exposed ($E$) and not feature in the cumulative count comprising $I + R + D$ epidemic states.

**FIGURE 9.**
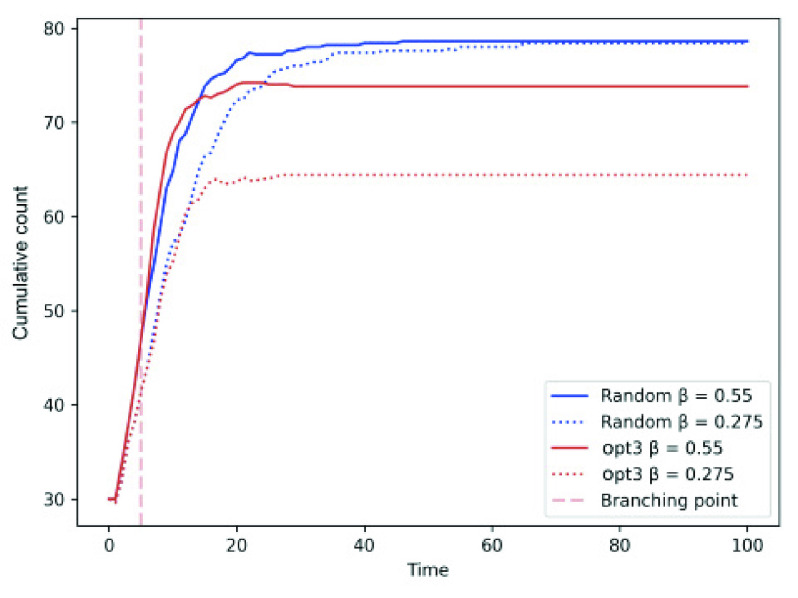
Cumulative count for varying contact rates $\beta = 0.275, 0.55$ in random and *opt-3* approaches.

*Contagion for Varying Contact Rates:* For two separate contact rate $\beta = 0.275, 0.55$, we compare the infection spread in *opt-3* against random individual mobility. Fig. 9 shows that *opt-3* exhibits a lower infection count than their random counterpart in the modified SEIRD model. On the other hand, for the modified SEIRD with branching timepoint set at $= 5$ minutes.

### D. Scalability

Let us analyze the performance of *opt-1*, *opt-2* and *opt-3* for larger populations. We consider population sizes of $50, 100, \cdots, 250$ and record the simulation time needed for the cumulative count of infected, recovered and dead individuals to reach 50% and 75% of the overall population sizes.

**FIGURE 10.**
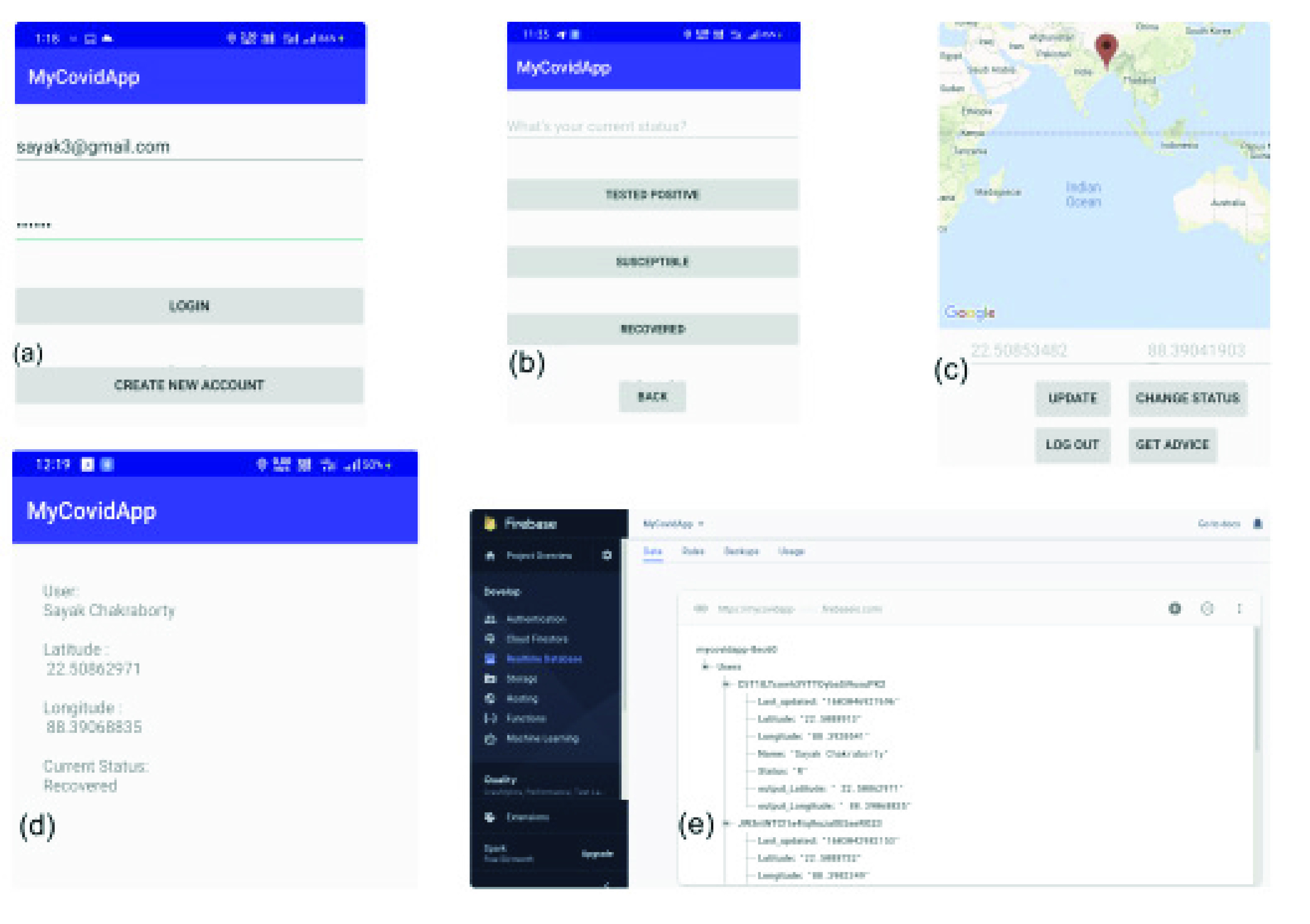
*MyCovid* App. User (a) signs up on the registration page, (b) enters epidemic status (susceptible, infected or recovered), (c) locations (through GPS), (d) summary of user information on the app, and (e) Firebase Realtime Database schema.

**FIGURE 11.**
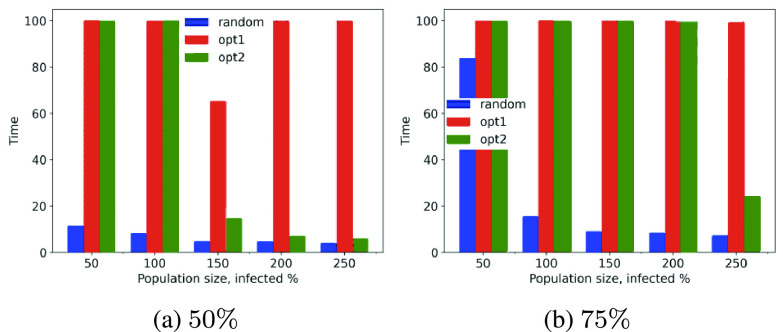
Time in minutes taken by the *opt-1*, *opt-2* and random to reach 50% and 75% cumulative count for variation in population sizes.

**FIGURE 12.**
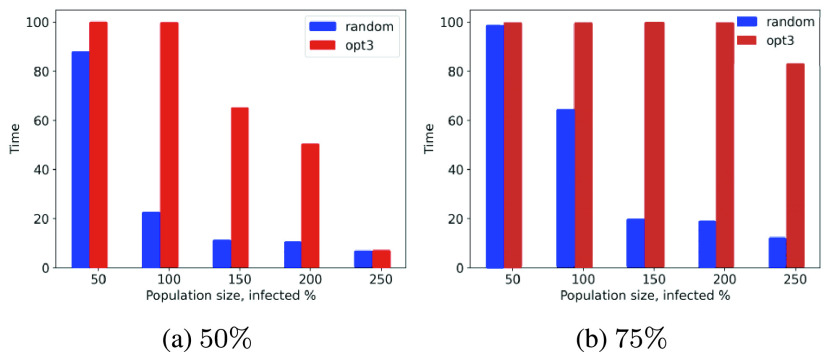
Time in minutes taken by the *opt-3* and random to reach 50% and 75% cumulative count for variation in the population sizes.

Scalability and computational cost are critical factors involving large population scenarios; we are trying to study this phenomenon with the *MyCovid* app (introduced in Sec. IV-F and Fig. 10). Experimentally, we show that social distancing strategies slow contagion down significantly as compared to the random counterparts. Figs. 11a, 11b, 12a and 12b show that while the random mobility results (blue bars) in cumulative count reaching 50% and 75% of total population in less than 25 minutes, the green and red bars corresponding to *opt-1*, *opt-2* and *opt-3* correspond to 100 minutes, suggesting that cumulative count does not reach 50% and 75% within the stipulated duration of 100 minutes.

### E. Human Mobility

**FIGURE 13.**
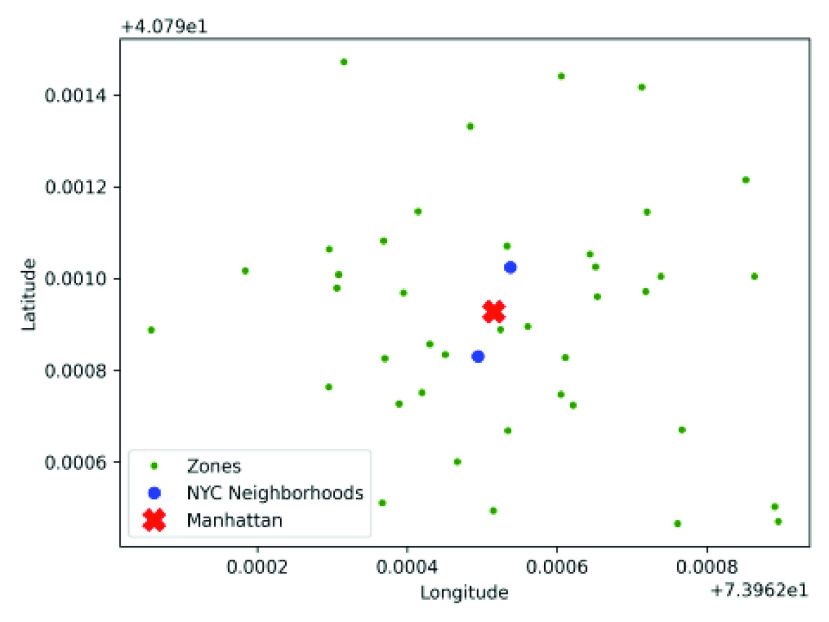
The 40 locations (represented as green dots) in and around the 2 neighborhoods (shown in blue) of Manhattan, New York City selected as cluster centers. Each person must be placed at one the 40 points and have 90% contact probability with other persons at the same location at the same time.

For the experiments on human mobility, we recreate the map of New York City, which comprises 5 boroughs – Manhattan, Bronx, Brooklyn, Queens and Staten Island. List of NYC boroughs and districts are taken from the official website of New York city [40] and the latitude and longitude of the 5 boroughs and 59 districts (or neighborhoods) are taken from the Python library for geocoding services, called GeoPy [41]. We select 40 locations (green dots in Fig. 13) in and around 2 neighborhoods (blue dots) selected as cluster centers from Manhattan, NYC (red cross). Distance between pair of points is calculated using geodesic distance function of GeoPy. An individual moves to any of the 40 locations on the map.

**FIGURE 14.**
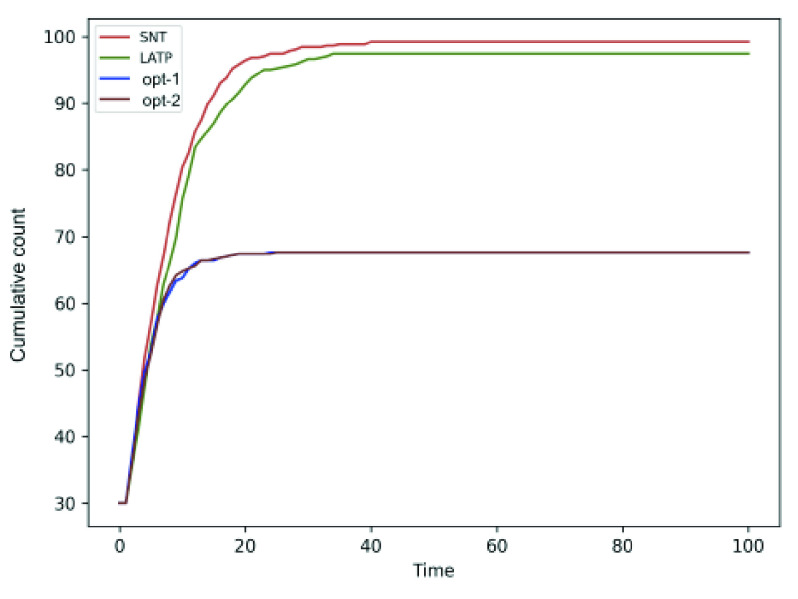
Comparison of the cumulative count for *opt-1* and *opt-2* against the SNT and LATP mobility models.

**FIGURE 15.**
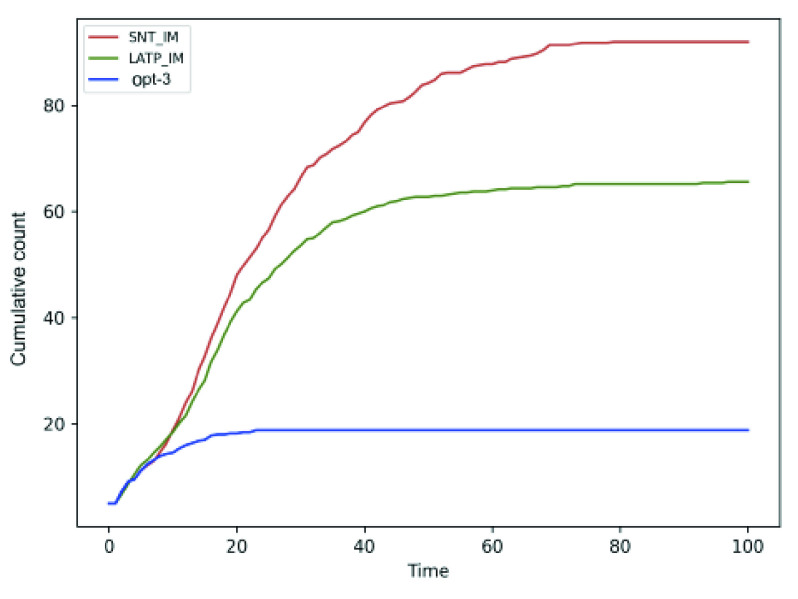
Comparison of the cumulative count for *opt-3* against the SNT and LATP mobility models.

We compare the performance of the social distancing optimization strategies against two human mobility models, namely LATP and SNT (discussed in Sec. II-D). Figs. 14 and 15 shows that the cumulative for SNT and LATP is significantly higher than that of the random mobility models. The SNT mobility, which relies on social interaction, requires a friendship graph with bidirectional link between friends of the same number of nodes as the total population (see Sec. II-D). We model the friendship graph as undirected Erdos-Renyi random graph [42] with edge probability $p = 0.1$. The results show that cumulative for the optimized mobility strategy, particularly *opt-3*, hit the plateau much earlier compared to the human mobility models. Overall, the proposed optimizations exhibit a notably lower rate of contagion than extant mobility approaches. It is worth mentioning that both SNT allows people to travel to waypoints with more social ties, while LATP causes individuals to prefers shorter trips. Either of these two conditions can result in notably higher social contact, necessitating the application of the proposed social distancing measures.

### F. MyCovid APP

*MyCovid App* is a mobile platform that constructs the temporal contact graph from the locations of the users and invokes one of the three optimization strategies (discussed in Sec. III-B–III-D) to inform their next location. Unlike the standard applications ([26]–[28]) discussed in Sec. I that construct a surveillance repository based on socio-demographic and medical characteristics, *MyCovid App* works at a granular level by minimizing the social ties between individuals that may potentially lead to spread of infection.

The user can register and use the fully functional *MyCovid App* by applying the following steps: (1) each user signs up on the registration page of *MyCovid*; (2) the app periodically transfers the last updated epidemic status (susceptible, infected or recovered) and location (lat-long coordinates through GPS) to the Firebase Realtime Database [43] – a cloud-hosted Google platform – connected to the server; (3) the server retrieves the information and runs the optimizer; (4) the respective new location recommended by the optimization is then sent back to each user. This location coordinate is not saved in the database, since there is no certainty that the user will follow the recommendation besides also reducing the load on the central server.

Fig. 10 shows the features of the *MyCovid* app. With express permission of the registered user, *MyCovid* creates a repository of user mobility data over time, which will serve as a testing set and help refine the proposed optimization strategies. It employs google API (fusedLocationProviderApi with settings of LocationRequest.PRIORITY_HIGH_ACCURACY and permission ACCESS_FINE_LOCATION) in the *high accuracy mode* that operates on WiFi/cellular in conjunction with GPS to minimize error in location tracking – a standard technology for location identification on android. One possible limitation of *MyCovid* is that the precision of locating smart devices can be 20 meters in worst case [44] (especially in indoor settings), although it is generally more accurate [45]. At present, *MyCovid* works on Android devices only, but we are working towards *MyCovid* on iOS platforms. We shared the source files for *MyCovid* app, with documentation and demonstrative video, on GitHub (https://github.com/satunr/ COVID-19/tree/master/Network%20Science) so that the network administrator can employ any location API of his choice to achieve the best results.

### G. Comparison of the Strategies

**FIGURE 16.**
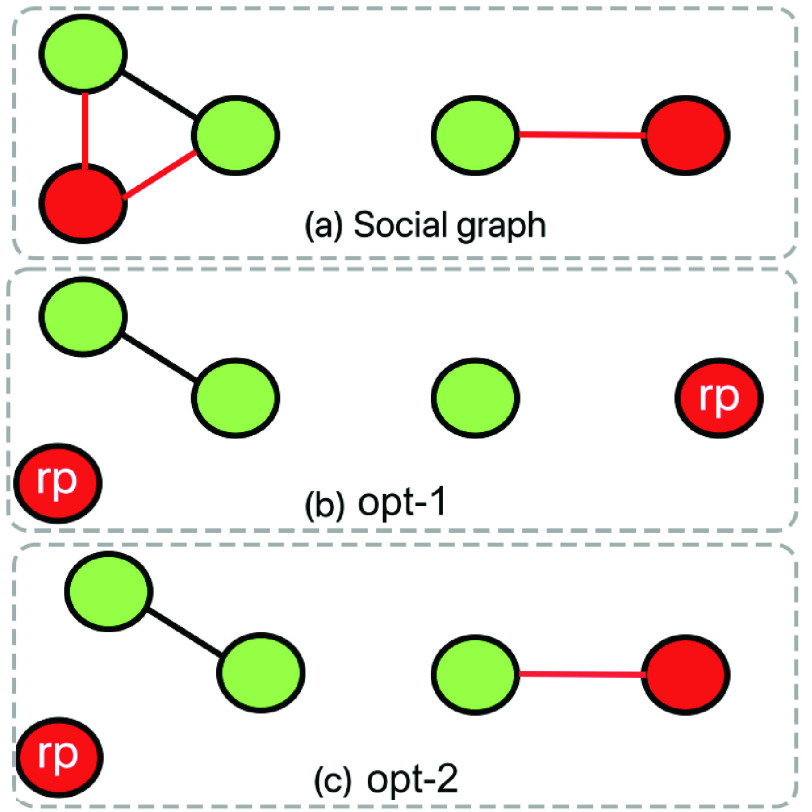
Comparison of *opt-1* and *opt-2* strategies (with susceptible and infected nodes colored green and blue, respectively). (a) input contact network, (b) *opt-1*, and (c) *opt-2*. The re-positioned individuals are labeled as *rp*.

The *MyCovid* app is customized to invoke any one of the three optimization strategies, and the choice of optimization is dependent on the underlying epidemic model as well as the contact network. As discussed in Sec. I-B, the *opt-1* and *opt-2* approaches both work on the SEIRD epidemic model. Fig. 11 shows that *opt-1* outperforms *opt-2* for most population sizes. This can be explained by considering a simple contact network shown in Fig. 16a, where the social ties that can cause contagion are marked red. The objective function of *opt-1* (Expression 10) tries to eliminate all red ties between susceptible and infected individuals (Fig. 16b). On the other hand, *opt-2* only eliminates red ties that belong to a cluster (Fig. 16c). It is worth noting that the *MyCovid* app may select *opt-2* (Expression 12) when there are multiple or large groups in the contact graph that have the triangles (discussed in Sec. II-B1) as their building blocks. Finally, we do not compare *opt-1* and *opt-2* against *opt-3* because unlike the first two, the latter operates on the modified SEIRD model where a part of the exposed population are perennially infectious. Also, *opt-3* is essentially a generalization of *opt-1* strategy, since it assumes that the infectivity of an individual is a continuous value between 0 and 1 (and not a binary like in *opt-1*).

### H. Effects of Flouting Recommendations

**FIGURE 17.**
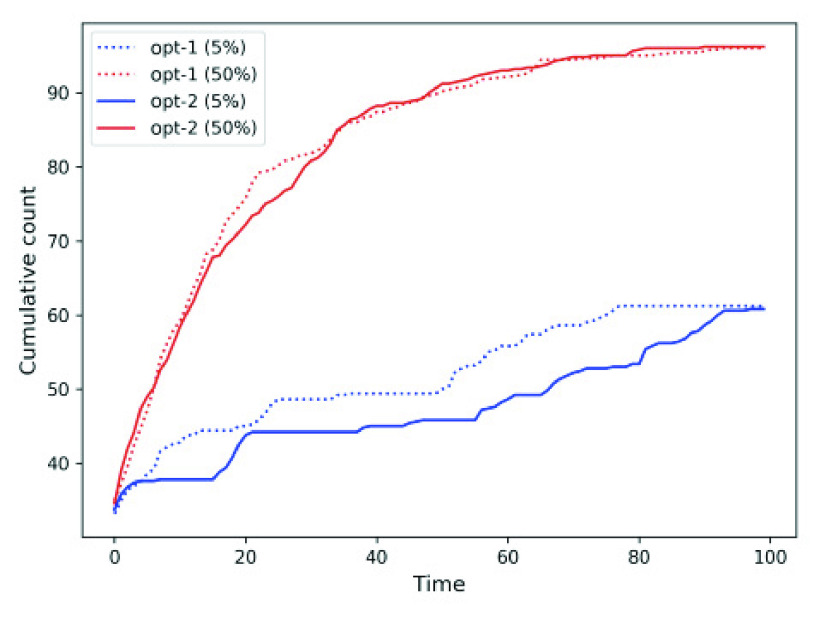
Cumulative count for *opt-1* and *opt-2* where people ignore the recommendation 5% and 50% of the times.

We know that the registered user of *MyCovid* app. are free to accept or reject the recommendation of the social distancing approaches. We study the cumulative count for two scenarios where each individual ignore the recommendation 5% and 50% of the times (and adopt random mobility) for *opt-1* and *opt-2*. Fig. 17 shows that, in either case, flouting the recommendations 50% of the times result in higher contagion manifested in overall increase in the cumulative count.

## V. Limitations of the Study

**FIGURE 18.**
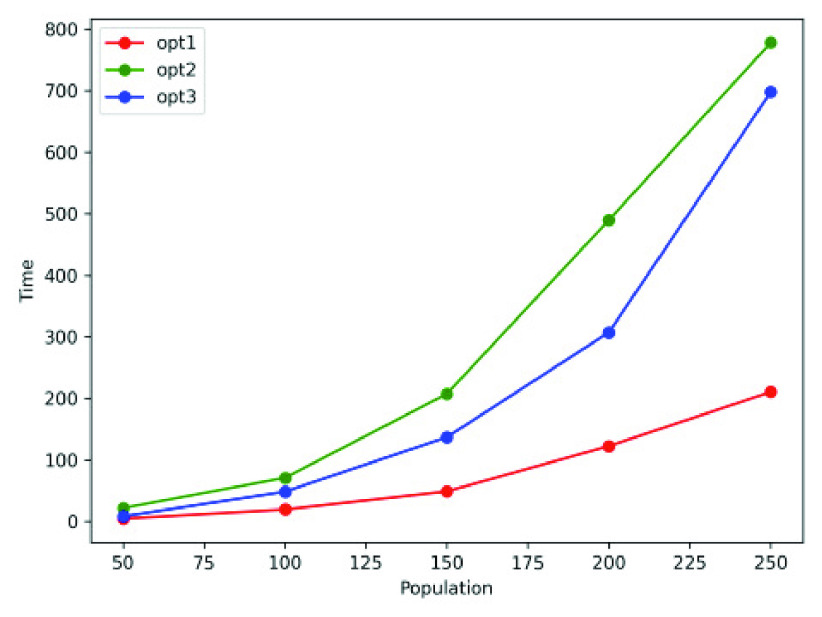
The running time (in seconds) of the three optimization strategies for population of 50 - 250 individuals.

One possible limitation in the proposed optimization strategies is the computational cost associated with scalability. In a population of $z$ individuals, the optimizer tunes $z$ parameters to achieve the socially distanced placement of individuals. To demonstrate this, we estimate the running time (in seconds) (for the optimization strategies) for a population of 50 - 250 individuals on a Mac OS Intel(R) Core(TM) i7-7820HQ CPU, 2.90GHz and 16 GM RAM system. Fig. 18 shows the near-exponential growth in the running time in seconds, necessitating scalable versions of these strategies in the future. We are in the early stages of designing greedy strategies that employ Markov Chain Monte Carlo methods and grid-based area partition to achieve time-efficient convergence.

## VI. Conclusion

In this work we leverage network science principles, namely network clustering and homophily to conceive network optimization strategies to enable socially distanced human mobility in two different SEIRD epidemic model scenarios. Based on current location, the optimizations recommend new locations of individuals, in order to minimize contact potentially resulting in contagion. We present a new measure of infectivity, termed contagion potential (CP), that addresses some of the challenges faced by the SEIRD model. Extensive simulation experiments show that our proposed approaches slow contagion better than several standard human mobility models. Finally, we present a mobile app., *MyCovid*, as a case study that employs one of the three strategies to inform mobility of registered users. With express permission from the user, the app will also build a repository of human mobility that would aid the future extensions of this work.

There are a few future directions in this line of work. First, we will adapt the greedy heuristics to different stages of the epidemic cycle. For instance, in the early stages of outbreak, the objective may be to enforce rigorous social distancing to clamp down on contagion. Conversely, during the later stages of the outbreak the restrictions may be gradually and strategically relaxed. Second, we are currently utilizing the *MyCovid* app to build a comprehensive repository of the mobility traces of the registered individuals that will be used to test the efficacy of the proposed approximation methods. Finally, we are going to explore the data-sharing and privacy concerns of *MyCovid* app. users. Different behavioral studies may emerge from the fact that users exhibit variable levels of adherence to the location recommendations of the app.

## Competing Interests

The authors have declared that no competing interests exist.
